# Whose health matters? Longitudinal analyses of older romantic couples’ health, physical capabilities, and sexual experiences

**DOI:** 10.1093/geronb/gbag060

**Published:** 2026-04-03

**Authors:** Yoobin Park, Säde Stenlund, Andrew Steptoe

**Affiliations:** Department of Psychiatry and Behavioral Sciences, University of California, San Francisco, San Francisco, California, United States; Department of Social and Behavioral Sciences, Harvard T. H. Chan School of Public Health, Boston, Massachusetts, United States; Unit of Public Health, University of Turku and Turku University Hospital, Turku, Finland; Department of Behavioural Science and Health, Institute of Epidemiology and Health Care, University College London, London, United Kingdom; (Social Sciences Section)

**Keywords:** Sexual well being, Sexual behavior, Successful aging, Frailty, Dyadic effects

## Abstract

**Objectives:**

While considerable work has linked sexual activity to health and well being in later life, the role of an individual’s physical health conditions in shaping their own and their partner’s later sexual experiences remains underexplored. This research examined concurrent and prospective dyadic effects of health and physical capabilities on sexual experiences.

**Methods:**

We used two-wave data from 1,301 heterosexual couples (*N *= 2,602) in the English Longitudinal Study of Ageing. Multilevel models were fitted, estimating the association of both partners’ self-rated health, grip strength, and gait speed with sexual interest, activity, and satisfaction concurrently and 4 years later. Various sociodemographic and psychosocial covariates were adjusted in addition to the baseline outcome levels in longitudinal models.

**Results:**

Both individuals’ and partner’s baseline self-rated health were positively related to intercourse frequency concurrently and at follow-up. Higher self-rated health was also associated with greater sexual satisfaction at follow-up across gender, but its positive associations with concurrent and later sexual interest were observed only among men. Men’s slower gait speed was linked to lower sexual interest for both partners at baseline and to women’s reduced sexual satisfaction at follow-up. Grip strength also showed some gender-specific associations with interest and intercourse frequency.

**Discussion:**

Sexual experiences in later life appear to be shaped by both partners’ physical health conditions, with notable gendered patterns. These findings underscore the importance of considering both individual and dyadic health factors in supporting sexual interest and activity in later life, as well as identifying objective markers that may precede declines in sexual health.

Sexual activity remains relevant and integral to most people’s lives across the life span (DeLamater, 2012; Træen & Villar, 2020). The maintenance of functional capacity, a central component of Rowe and Kahn’s (1997) conceptualization of successful aging, may both shape and be shaped by sexual well being. However, sexual aspects of later life continue to be an “overlooked aspect of successful aging” (Soysal & Smith, 2022, p. 1235).

A growing body of evidence highlights the implications of sexual activity for quality of life, including physical well being. For example, prospective studies have linked sexual activity to a lower incidence of cardiovascular disease (Teng et al., 2024) and a reduced risk of mortality (Cao et al., 2020). At a daily level, engaging in sexual activity has been related to better sleep quality and lower blood pressure the next morning (Park et al., 2024). That said, the link between sexual activity and physical health is likely bidirectional (Gianotten et al., 2021); although sex may promote health, better health conditions may also increase desire for sexual activity and enable individuals to engage in it (Erens et al., 2019).

There is indeed considerable cross-sectional evidence suggesting that healthy individuals are more sexually active (Field et al., 2013; Freak-Poli et al., 2017; Lindau & Gavrilova, 2010; Træen et al., 2018). Yet, longitudinal research on whether better physical health tends to precede later sexual experiences is scarce. More broadly, research on older individuals’ sexuality and health has been notoriously limited to cross-sectional investigations (DeLamater, 2012; Gillespie et al., 2017), despite the conceptual models (i.e., biopsychosocial perspectives on late-life sexuality; DeLamater & Koepsel, 2015) acknowledging physical weakness as a *predictor* of changes in sexual experiences. One exception is a recent study among Czech older adults that examined chronic health conditions as a predictor of sexual activity status—specifically, whether one is sexually active across 2 years, as opposed to (a) being consistently sexually inactive, or (b) ceasing to be sexually active at follow-up (Gore-Gorszewska et al., 2024). They found that those with fewer self-reported chronic conditions at baseline were more likely to be in the consistently sexually active group. Although the meaning of this aggregate score is somewhat ambiguous, another study among older men that examined independent associations of various chronic health conditions with sexual activity status found that diagnoses of diabetes in particular predicted later inactivity (Hyde et al., 2010).

Given that most sexual interactions occur within established romantic relationships, with a regular partner (Træen et al., 2018), sexual interest and activity, as well as overall evaluation of sex lives, may be shaped not only by the person’s own but also by their partner’s health conditions (Bell et al., 2017). From the perspective of successful sexual aging (Štulhofer, 2025), a partner’s disinterest in or inability to engage sexually can constitute an external constraint on sexual expression that shapes sexual trajectories in later life. Empirically, a partner’s physical limitations have been identified as a common reason for sexual inactivity, particularly among women (Lindau et al., 2007). Reports of a partner’s illness that causes sexual problems have also been linked to women’s (but not men’s) lower frequency of sexual intercourse (Kontula & Haavio-Mannila, 2009). Together, these findings appear to suggest that women’s sexual experiences may be particularly sensitive to partner health. However, this gendered pattern is not consistently supported across studies. One study of older men showed that men who reported having a partner with physical limitations were also less likely to be sexually active and to remain sexually active approximately 6 years later (Hyde et al., 2010). Another study found that, across gender, poorer perceptions of a partner’s health were associated with greater odds of sexual inactivity, even after adjusting for individuals’ perceptions of their own health (Karraker & Delamater, 2013). Notably, one study reported that poorer perceived spousal health was associated with *lower* odds of reporting an inability to maintain a sexual relationship, despite showing the expected positive association with odds of sexual dissatisfaction; these effects were independent of individuals’ own health, and gender differences were not tested (Syme et al., 2013).

While promising, previous work has been limited in its ability to precisely capture the *dyadic* interplay of health and sexual experiences, in part because most studies rely on one partner’s reports (cf. Galinsky & Waite, 2014), which may be biased for various reasons, including motivated reasoning (e.g., attributing sexual inactivity to a partner’s characteristics rather than oneself) or projection (e.g., projecting one’s own physical limitations onto the partner). Further, the narrow coverage of health indicators and sexual experiences in prior work has constrained a fuller understanding of how physical health conditions predict later sexual experiences. Finally, inconsistent findings and, in some cases, a lack of formal tests regarding gender differences highlight a persistent gap in understanding whether and how these associations differ for men and women.

Addressing these gaps, the present research uses dyadic data from older romantic couples to examine how *both partners’* characteristics jointly contribute to their current or later sexual interest, activity, and satisfaction. In addition to partners’ subjective evaluations of their health, we examined their physical capabilities, objectively assessed through standard strength tests. Specifically, we used grip strength and gait speed, both of which commonly serve as indicators of physical capabilities (Cooper et al., 2011). Grip strength is widely used as a marker of upper-body muscle strength and overall vitality, whereas gait speed reflects the integrated functioning of multiple physiological systems, including lower-body strength, balance, and cardiorespiratory fitness. Both have been linked with important health outcomes, including mortality risk (Elbaz et al., 2013; Xie et al., 2022).

In short, our research addressed the following question: how are individuals’ and their partners’ self-rated health and physical capabilities related to sexual experiences (i.e., sexual interest, sexual activity status, frequency of intercourse, and sexual satisfaction) concurrently and 4 years later? We also examined if and how these associations differed for men and women. Although this study was not preregistered, several of our research questions were conceptual replications of prior work and informed a priori expectations. Specifically, we expected that individuals’ own and their partner’s self-rated health would show concurrent positive associations with greater odds of being sexually active and having more frequent intercourse (e.g., Karraker & Delamater, 2013). In contrast, we did not advance specific predictions regarding physical capabilities or prospective associations.

## Method

### Participants and procedure

Data for this research came from the English Longitudinal Study of Ageing (ELSA), an ongoing study that began in 2002, based on the English population aged 50 and older living in private households. For information on data access, please see https://www.elsa-project.ac.uk/accessing-elsa-data. At Wave 1, 12,099 individuals, which included age-eligible core members and their spouses who did not need to meet the age criteria, were recruited. ELSA interviews participants every 2 years, collecting rich information on their health (emotional, cognitive, physical) as well as their social and economic circumstances. For more information on the study and the cohort, please see Steptoe et al., 2013. The current analysis focuses on data collected in 2012/2013 (Wave 6) and 2016/2017 (Wave 8), when the sexual activity module was administered. The total number of respondents in Waves 6 and 8 was 10,601 and 8,445, respectively; of these, 85% and 86% completed the self-completion questionnaires. Note that although these data have been used in prior work (e.g., Smith et al., 2020), none have addressed our research question or, importantly, any dyadic effects.

Our analytic sample included all individuals who completed the sexual activity module in both waves and whose partner also did, with the partner being the same person across waves. Given our interest in the gender effects, we focused on opposite-sex couples, which resulted in the exclusion of 24 couples. The final sample included 1,301 individuals and their partners (total *N *= 2,602). Participants were 63.3 years old on average (*SD *= 8.1) and were predominantly White (98%; all but 53). More than half of the sample (58%; *n *= 1,383) reported having completed upper secondary education, and 24% (*n *= 575) tertiary education. Most (94%; *n *= 2,448) were married. The median total family wealth (based on the harmonized variable, *h6atotb*, a household-level measure aggregating housing, financial, business, and other assets) was £303,000 (mean = 450,164; standard deviation = 879,820).

#### Analytic sample comparison

To examine if and how our sample selection biased the type of individuals we analyzed, we compared those who were included in our primary analyses (*n *= 2,602 who had dyadic data on sexual modules at both time points) against partnered individuals who were not (*n *= 2,492) in terms of various sociodemographic (age, education, wealth), psychosocial (depression, life satisfaction, relationship closeness), and health-related variables (number of chronic conditions, physical activity). We found that those included in our analyses were younger (*p* < .001) but were not any more likely to be highly educated (*p* = .19) or in the wealthiest tertile (*p* = .99). They felt closer to their partner (*p* = .003) but did not differ in life satisfaction (*p* = .06) or depression (*p* = .09). Finally, they had fewer chronic conditions (*p* < .001) and self-reportedly engaged in all types of exercise more frequently (*p*s < .01). Full statistics are reported in Supplementary Table 1.

### Measures: sexual experiences

#### Sexual interest

At baseline, participants responded to the following question: “How often did you think about sex during the past month? This includes times of just being interested in sex, daydreaming or fantasizing about sex, as well as times when you wanted to have sex.” The response options ranged from 1 (*not at all*) to 7 (*more than once a day*). At follow-up, the same question was asked with the stem changed to “during the past 12 months.” The response options ranged from 1 (*not at all*) to 6 (*once a day or more*). For consistency across waves, we recoded the baseline 7-point scale to match the 6-point scale used at follow-up, combining option 7 with option 6 (originally *once a day*).

#### Sexual activity and frequency

Participants were asked if they had had any sexual activity (sexual intercourse, masturbation, petting, or fondling) in the past year (Yes/No; sexual activity status). Those who responded with a “Yes” were directed to further questions, including “how many times have you had or attempted sexual intercourse [vaginal, anal, or oral sex]” (intercourse frequency). As in sexual interest, this question referred to the past month at baseline and the past 12 months at follow-up. Response options ranged from 1 (*not at all*) to 7 (*more than once a day*) at baseline and from 1 (*not at all*) to 6 (*once a day or more*) at follow-up. As with sexual interest, baseline responses were harmonized to the 6-point follow-up scale by recoding option 7 to 6. Participants who reported no sexual activity were coded as 1 (*not at all*) for intercourse frequency.

#### Sexual satisfaction

At baseline, participants who indicated being sexually active were asked how satisfied they had been with their overall sex lives during the past 3 months. At the follow-up, this question was asked with reference to the past 12 months. Response options ranged from 1 (*very satisfied*) to 5 (*very dissatisfied*) and were recoded so that higher values indicate higher satisfaction.

### Measures: health and physical capabilities

#### Self-rated health

Participants answered the question, “Would you say your health is…” using a scale ranging from 1 (*excellent*) to 5 (*poor*). We recoded this item so that higher values indicate better health.

#### Grip strength (kg)

During a nurse visit, participants completed a grip strength module in which they were asked to squeeze a Smedley hand‐held dynamometer as hard as possible for a couple of seconds. This process was repeated three times for each hand. We used the average of the three measurements from the dominant hand for our analyses (Smith et al., 2019).

#### Gait speed (m/s)

At baseline, participants aged 60 or older were asked to walk, with or without a gait-assistance device, 2.44 m at their usual walking pace, and the time from start to finish was recorded. The test was repeated twice. We used the average of the two walks in our analyses (Hackett et al., 2018). Following previous research, we created a binary indicator of slowness, defined as ≤0.8 m/s, considered to indicate low physical performance (De Souza et al., 2025). Out of 1,676 individuals who completed the test, 13% were coded as slow walkers (103 men, 113 women). We also report full results from models using gait speed as a continuous measure in Supplementary material.

### Covariates and missing values

We controlled for sociodemographic characteristics including participants’ gender, participants’ and their partner’s age, race (White vs. non-White), education (<less than upper secondary, upper secondary and vocational training, tertiary), and total household wealth (tertiled). Socioeconomic status, such as educational attainment, has been linked with both health and sexual activity in later life (Ikeda, 2024; Momtaz et al., 2014) and was therefore considered important to account for. We also controlled for participants’ marital status (married vs. not), relationship closeness (assessed using one item, “How close is your relationship with your spouse or partner?” on a 4-point scale), and depressive symptoms (assessed using the 8-item Center for Epidemiological Studies-Depression scale; Radloff, 1977), all of which could serve as potential confounders (Roman Lay et al., 2023; Skoblow et al., 2023). Missing values for covariates were minimal and below 2%, except for educational attainment (8%), and were imputed using a random forest-based imputation algorithm (Stekhoven & Bühlmann, 2012). Our key variables (self-rated health, physical capabilities, and sexual experiences) were not imputed.

### Analysis plan

All analyses were conducted in R. To account for the nested structure of the data, we conducted multilevel modeling using the *lme4* package (Bates et al., 2015). We used an Actor–Partner Interdependence Model (Kenny et al., 2006) to examine both actor and partner effects—that is, how one’s own health or physical capabilities (actor effect), as well as their partner’s health or physical capabilities (partner effect), are related to their sexual experiences. We first examined concurrent associations at baseline, followed by prospective associations in models that included baseline level of each outcome as a covariate (e.g., baseline sexual interest was controlled for in models predicting follow-up interest). We also tested gender interactions and dropped nonsignificant interaction terms. Any significant interactions were probed by estimating simple slopes for men and women using the *emmeans* package (Lenth, 2022). Statistical inference was based on a significance threshold of *p* < .05. Given the exploratory nature of the analyses and the treatment of each model as an independent test, we did not apply corrections for multiple comparisons and report uncorrected *p*-values while acknowledging the increased potential for Type 1 error (Rubin, 2021).

#### Robustness check

Considering that baseline sexual dysfunction may confound any association between health or physical capabilities and later sexual experiences, we re-ran our prospective analyses, excluding couples with men who were “never able to get and keep an erection which would be good enough for sexual activity” at baseline, which resulted in the exclusion of 193 couples. All reported results remained unchanged in this analysis.

## Results

### Preliminary analyses

At baseline, 81% of our participants were sexually active, and 89% of them remained active at follow-up. A transition matrix illustrating changes in sexual activity across waves is presented in Supplementary Figure 1. Table 1 presents descriptive statistics and correlations for key study variables at baseline, separately for men and women.

**Table 1:** Descriptive statistics and zero-order correlations between key study variables at baseline.

Variable	Men							
	1	2	3	4	5	6	7	Men *M* (*SD*)
**Women**								
1. Self-rated health	**.41** *	.15*	.38*	.19*	.16*	.20*	.15*	3.39 (1.05)
2. Grip strength	.23*	**.26** *	.30*	.25*	.20*	.19*	.08*	29.30 (6.66)
3. Gait speed	.41*	.27*	**.48** *	.23*	.20*	.16*	.01	1.00 (0.17)
4. Sexual interest	.14*	.15*	.19*	**.42** *	.53*	.44*	.01	4.42 (1.60)
5. Sexual activity status	.18*	.11*	.18*	.50	**84%**	.41*	—	0.84 (0.36)
6. Intercourse frequency	.15*	.15*	.14*	.55*	.51*	**.81** *	.37*	2.31 (1.38)
7. Sexual satisfaction	−.02	.08	−.03	.16	—	.29*	**.36** *	3.58 (1.19)
**Women *M* (*SD*)**	3.44(1.05)	17.77(4.63)	0.97(0.16)	3.10(1.52)	0.77(0.42)	2.28(1.37)	3.79(1.09)	

*Note*. Men’s and women’s correlations, means (*M*), and standard deviations (*SD*) appear above and below the diagonal, respectively. Bolded diagonal values represent within-dyad correlations (percent agreement for sexual activity status). Continuous gait-speed score is used. Sexual activity status is coded as 0 = inactive, 1 = active. Sexual satisfaction was only assessed among participants who reported being sexually active.

* *p* < .05.

### Cross-sectional analyses

Table 2 shows a summary of the results from cross-sectional models across outcomes. Full results with all covariates can be found in Supplementary material.

**Table 2:** Self-rated health and physical capabilities associated with sexual outcomes at baseline.

Predictors	Sexual interest			Activity			Intercourse			Satisfaction		
	*b*	*SE*	*p*	*OR*	*SE*	*p*	*b*	*SE*	*p*	*b*	*SE*	*p*
**Self-rated health (*Ns*: 2,595, 2,596, 2,584, 1,619)**												
Actor	0.17	0.04	<.001	1.87	0.35	.001	0.09	0.02	<.001	0.17	0.04	<.001
Partner	−0.02	0.04	.623	1.02	0.19	.921	0.08	0.02	.001	−0.03	0.04	.522
Actor × Gender	−0.14	0.06	.018							−0.25	0.06	<.001
Partner × Gender	0.08	0.06	.173							0.14	0.06	.021
**Grip strength (*N*s: 1,786, 1,783, 1,776, 1,060)**												
Actor	0.03	0.01	<.001	1.04	0.04	.400	0.01	0.01	.079	0.01	0.01	.087
Partner	0.00	0.01	.988	0.98	0.04	.650	0.01	0.01	.093	0.00	0.01	.462
Actor × Gender	−0.03	0.01	.035									
Partner × Gender	0.01	0.01	.599									
**Slowness (*N*s: 1,401, 1,400, 1,392, 744)**												
Actor	−0.54	0.19	.004	0.55	0.19	.089	−0.02	0.09	.818	−0.04	0.14	.784
Partner	0.12	0.17	.488	0.70	0.25	.305	−0.09	0.09	.321	0.11	0.14	.443
Actor × Gender	0.50	0.25	.048									
Partner × Gender	−0.52	0.25	.039									

*Note. b* = unstandardized coefficient; *OR* = odds ratio. All models adjusted for sociodemographic, relationship, and psychological covariates. Gender interactions were retained if the interaction with either actor or partner effect was significant. Standard errors for sexual activity refer to the log-odds estimates. *N*s correspond to the number of individuals included in each model. Full results are reported in Supplementary material.

#### Self-rated health

**Figure 1:**
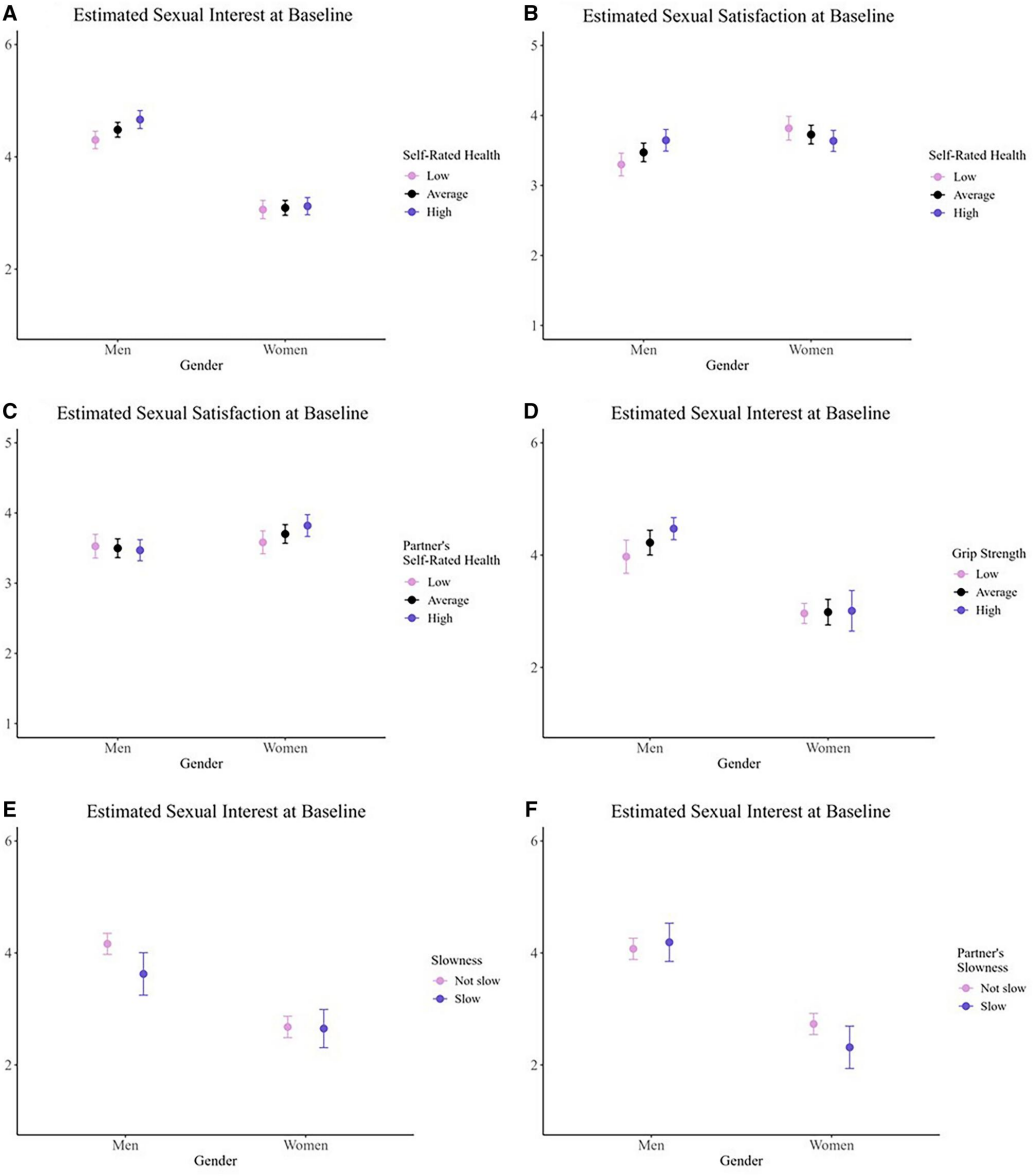
Gender interactions with self-rated health and physical capabilities associated with sexual experiences at baseline.

Across genders, higher self-rated health was associated with greater odds of being sexually active and having more frequent intercourse. Having a partner with higher self-rated health was independently associated with having more frequent intercourse. However, associations between self-rated health and sexual interest or satisfaction varied by gender. Among men, higher self-rated health was associated with greater sexual interest, *b *= 0.17, *t *= 4.09, *p* < .001 (Figure 1A), and greater sexual satisfaction, *b *= 0.17, *t *= 3.90, *p* < .001 (Figure 1B). Among women, self-rated health was not associated with interest, *b *= 0.03, *t *= 0.69, *p* = .49 (Figure 1A), and in fact, associated with lower sexual satisfaction, *b *=* −*0.09, *t *=* −*2.02, *p* = .04 (Figure 1B). Gender differences also emerged for partner effects on sexual satisfaction: For women, having a partner with higher self-rated health was associated with greater sexual satisfaction, *b *= 0.11, *t *= 2.68, *p* = .008, but this partner effect was not significant for men, *b *=* −*0.03, *t *=* −*0.64, *p* = .52 (Figure 1C).

#### Grip strength

One gender interaction emerged with grip strength, suggesting that greater grip strength was associated with greater sexual interest among men, *b *= 0.03, *t *= 4.06, *p* < .001, but not among women, *b *= 0.003, *t *= 0.28, *p* = .78 (Figure 1D).

#### Gait speed

Significant gender interactions suggested that men’s slowness was associated with reduced sexual interest for both partners. Specifically, men’s own slowness was associated with their lower sexual interest, *b *=* −*0.54, *t *=* −*2.90, *p* = .004, whereas no such actor effect emerged for women, *b *=* −*0.03, *t *=* −*0.21, *p* = .83 (Figure 1E). In contrast, partner’s slowness was unrelated to interest among men, *b *= 0.12, *t *= 0.69, *p* = .49, but was associated with lower interest among women, *b *=* −*0.41, *t *=* −*2.20, *p* = .03 (Figure 1F).

In sum, higher self-rated health, and to some degree, having a partner with higher self-rated health, were linked to being more sexually active across gender. However, associations with sexual interest and satisfaction differed by gender, with positive actor effects evident only among men, and women’s satisfaction linked instead to partner self-reported health. Further, men’s physical capabilities, reflected in greater grip strength and the absence of slowness, were positively associated with their own and their partner’s sexual interest.

### Prospective associations

Table 3 shows a summary of the results from longitudinal models across outcomes, with full results available in Supplementary material. Note that each model adjusted for an equivalent outcome assessed at baseline.

**Table 3:** Self-rated health and physical capabilities associated with sexual outcomes at follow-up.

Predictors	Sexual interest			Activity			Intercourse			Satisfaction		
	*b*	*SE*	*p*	*b*	*OR*	*p*	*b*	*SE*	*p*	*b*	*SE*	*p*
**Self-rated health (*N*s = 2,566, 2,595, 2,570, 1,603)**												
Actor	0.12	0.03	<.001	1.29	0.12	.005	0.05	0.02	.002	0.08	0.03	.004
Partner	0.02	0.03	.577	1.23	0.11	.021	0.04	0.02	.014	0.02	0.03	.507
Actor × Gender	−0.12	0.04	.007									
Partner × Gender	−0.03	0.04	.526									
**Grip strength (*N*s = 1,767, 1,782, 1,767, 1,049)**												
Actor	0.00	0.00	.442	0.99	0.02	.789	0.00	0.00	.373	−0.00	0.01	.826
Partner	−0.00	0.00	.770	1.01	0.02	.448	0.02	0.01	.001	0.00	0.01	.612
Actor × Gender							0.01	0.01	.497			
Partner × Gender							−0.02	0.01	.035			
**Slowness (*N*s = 1,382, 1,399, 1,382, 733)**												
Actor	0.03	0.09	.741	0.70	0.18	.178	−0.00	0.06	.960	−0.31	0.18	.086
Partner	0.11	0.09	.235	0.91	0.24	.714	−0.08	0.06	.202	0.10	0.16	.529
Actor × Gender										0.42	0.24	.079
Partner × Gender										−1.16	0.25	<.001

*Note. b* = unstandardized coefficient; *OR* = odds ratio. All models adjusted for baseline levels of the outcome, as well as sociodemographic, relationship, and psychological covariates. Gender interactions were retained if the interaction with either the actor or partner effect was significant. Full model results are provided in Supplementary material.

#### Self-rated health

**Figure 2:**
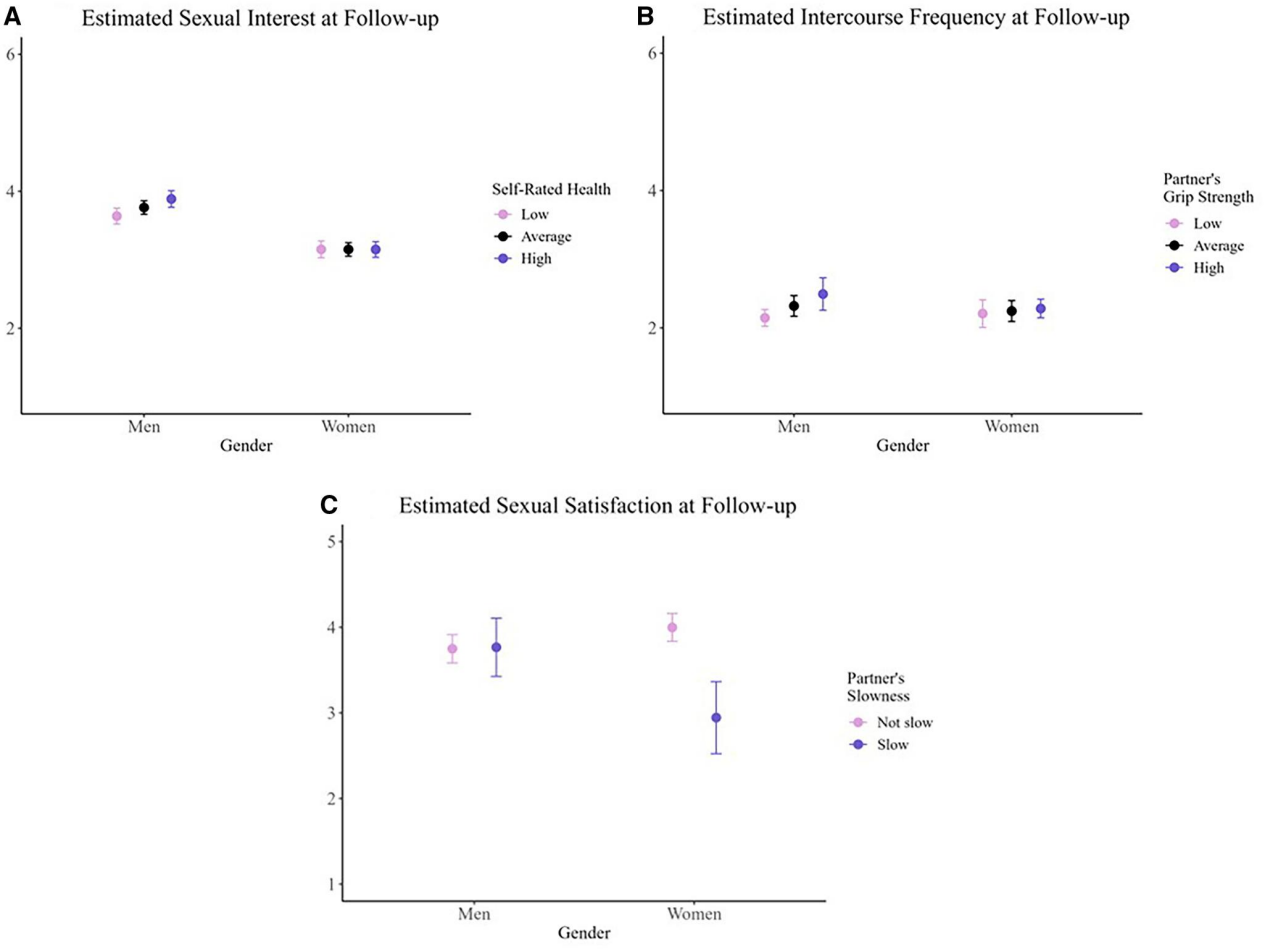
Gender interactions with self-rated health and physical capabilities associated with sexual experiences at follow-up levels (adjusting for baseline).

Across gender, higher baseline levels of both self-rated health and partner’s self-rated health were associated with greater odds of being sexually active and reporting more frequent intercourse at follow-up. Higher self-rated health was also associated with greater sexual satisfaction at follow-up. However, the association with sexual interest varied by gender such that higher self-rated health was associated with increased interest among men, *b *= 0.12, *t *= 3.71, *p* < .001, but not women, *b *=* −*0.001, *t *=* −*0.02, *p* = .98 (Figure 2A).

#### Grip strength

One significant gender interaction emerged for the partner effect, indicating that for men, having a partner with greater grip strength was associated with more frequent intercourse at follow-up, *b *= 0.02, *t *= 3.26, *p* = .001. No such effect was observed among women, *b *= 0.004, *t *= 0.91, *p* = .36 (Figure 2B).

#### Gait speed

One significant gender interaction suggested that partner’s baseline slowness was associated with lower sexual satisfaction among women, *b *=* −*1.06, *t *=* −*5.53, *p* < .001, but not among men, *b *= 0.10, *t *= 0.63, *p* = .53 (Figure 2C).

In sum, both individuals’ and their partners’ higher self-rated health appeared conducive to sustaining sexual activity, including intercourse. Higher self-rated health at baseline was also associated with increased sexual satisfaction across gender, although, as in the cross-sectional findings, its implication for interest was observed only among men. There was also evidence that women’s greater grip strength was associated with men reporting more frequent intercourse, whereas men’s slowness was associated with women reporting reduced sexual satisfaction.

## Discussion

Using dyadic data of older romantic couples, we examined the concurrent and 4-year longitudinal associations of both partners’ self-rated health and physical capabilities with their sexual interest, activity, and satisfaction. Overall, our findings support calls to move beyond an individual-focused perspective toward a dyadic perspective in understanding older adults’ sexual experiences (Waite et al., 2017). Given that most partnered sexual interactions occur within established relationships (Træen et al., 2018), both one’s own and the partner’s health conditions appear to play a role in predicting sexual experiences. For example, higher self-rated health in *both* partners was independently associated with more frequent intercourse at baseline, as well as with greater odds of remaining sexually active and increases in intercourse frequency over 4 years. Across outcomes, the significant prospective associations were small-to-modest in magnitude, partly reflecting adjustment for baseline levels of the corresponding outcome. For self-rated health, odds ratios for sexual activity were 1.29 for actor and 1.23 for partner effects, indicating 29% and 23% greater odds of being sexually active at follow-up for a one-unit difference in one’s own and partner’s self-rated health. Given the high prevalence of sexual activity, these odds ratios correspond to small differences in probability; unstandardized coefficients for continuous outcomes were similarly small in absolute magnitude.

Our findings revealed several gendered patterns, particularly for sexual interest. At baseline, higher self-rated health, grip strength, and absence of slowness were related to greater interest among men, but none of these actor effects were significant for women. Instead, men’s slowness was associated with lower interest among women, a partner effect not observed in men. Likewise, the association between higher self-rated health and increased interest 4 years later was evident only among men. Together, these findings suggest that men’s sexual interest is more closely tied to their own health and capabilities than women’s is. This aligns with the literature on women’s sexual interest, which does acknowledge the role of physical conditions but places great emphasis on psychological or relational factors (e.g., Cherkasskaya & Rosario, 2019; Meana, 2010). The significant partner effect observed only for women’s interest also reinforces the view that women’s sexual interest may be responsive to relational factors and is consistent with previous work. For example, men’s successful aging (a latent construct capturing emotional and social well being) has been associated with women’s changes in sexual desire, but not vice versa (Štulhofer et al., 2019); likewise, stronger evidence is found for men’s feelings of intimacy predicting their partner’s sexual well being than the reverse (Štulhofer et al., 2020), suggesting that women’s subjective sexual experiences may be more sensitive to their partner’s conditions than men’s are.

In contrast, fewer gender differences emerged for sexual activity or intercourse frequency and changes therein. Only one gender difference emerged with a partner effect of grip strength, whereby women’s strength was associated with men’s reports of intercourse frequency, but not vice versa. All other effects were comparable across gender. For both men and women, better self-rated health was associated with greater odds of being and remaining sexually active as well as more frequent intercourse at both baseline and follow-up. Independently, having a partner with higher self-rated health was associated with remaining sexually active by the follow-up, and with greater intercourse frequency both concurrently and longitudinally. Thus, unlike interest in sexual experiences, which may be shaped differently for men and women, the extent to which partners’ physical conditions are tied to sexual behavior appears more generalizable across genders.

Looking at the global evaluation of sexual life, which reflects both sexual interest and actual engagement in sexual activity, one particularly notable finding was that women’s sexual satisfaction was higher when their partner reported better health, but, if anything, lower when women themselves reported better health. Although healthier women may engage in more frequent intercourse, as suggested by the association between self-rated health and intercourse frequency in our data (Table 2), frequency alone may be insufficient to ensure greater satisfaction. Especially in heterosexual relationships, women are less likely than men to consistently experience orgasm (McElroy & Perry, 2024), and sexual activity that is not fulfilling may, in turn, lower sexual satisfaction. From this perspective, it may be the partner’s health that is more critical for ensuring the quality, not merely the quantity, of women’s sexual experiences.

Our findings linking physical health status to later sexual activity are important because the pathway from health to sexual engagement has received comparatively less attention than the growing body of work examining sexual activity as a predictor of health outcomes. In fact, in our analyses testing this reverse pathway (i.e., whether sexual experiences predicted health outcomes 4 years later), there was no evidence of the putative “benefits” of sexual activity (see the Supplementary material for the full results). The only exception was a gender interaction indicating that, among men, higher sexual interest at baseline was associated with increases in grip strength at follow-up. Even in cases when sexual activity may indeed confer physical health benefits, failing to consider that individuals’ and their partners’ health conditions may act as antecedents of sexual activity risks overestimating the causal influence of sexual activity on health. For example, linking sexual activity at a single time point to distal outcomes such as mortality (Cao et al., 2020; Teng et al., 2024) often implies a unidirectional causal pathway, yet such associations may partly reflect preexisting individual or dyadic health conditions. Although the individual’s own health is sometimes statistically controlled in this type of work, the role of partner health is rarely accounted for, despite emerging evidence that partner characteristics can meaningfully contribute to individual health outcomes (Burns, 2020; Stavrova, 2019). Our findings highlight the importance of considering both actor and partner characteristics in modeling the presumed bidirectional link between health and sex. It is likely that sexual activity has short-term health-promoting effects (e.g., energy boosts), which may both accumulate to support long-term health and also serve to sustain sexual engagement, potentially creating a positive feedback loop within couples.

Our work extends previous investigations linking physical health conditions to sexual experiences by exploring both subjective perceptions of health and objective indicators of physical capabilities, examining dyadic effects, and conducting prospective analyses, all of which are rare in studies of older couples’ sexuality. In particular, this work revealed the potential implications of gait speed for sexual experiences for the first time—specifically, men’s physical slowness was associated with both their own and their partners’ lower sexual interest, as well as their partners’ decreases in satisfaction. It is conceivable that gait speed, reflecting lower-body strength, coordination, and mobility, may have unique implications (e.g., positioning and movement) for the quality and feasibility of various sexual activities from the partner’s perspective. Alternatively, men’s slowness may serve as a visible, salient indicator of declining vitality, prompting women to recalibrate their expectations or investment in sex (e.g., by raising concerns about potential health risks of sexual activity), which could also contribute to lower interest and satisfaction. We also observed more limited associations involving grip strength: Men’s baseline grip strength was associated with their higher concurrent sexual interest, whereas women’s baseline grip strength was associated with their male partner’s greater intercourse frequency 4 years later. The lack of associations with women’s sexual experiences suggests that women’s sexual experiences may be more responsive to partners’ visible indicators of vitality than to the underlying physical capacity that grip strength captures (Bohannon, 2019).

While these findings, particularly the gendered associations, warrant replication, they highlight the value of assessing physical vitality, such as gait speed and grip strength, when studying sexual experiences in later life. Future research should explore other markers of vitality and uncover those that best predict the maintenance of sexual activity and satisfaction. Such work could offer valuable insights for identifying individuals at greater risk of declining sexual health—individuals who may benefit from open communication around these issues, a topic often overlooked by healthcare professionals but worthy of greater attention (Dyer & das Nair, 2013).

Several limitations of this study should be noted. Although large, our sample lacked ethnic diversity (e.g., 98% White) and was geographically homogeneous (England). Because gendered patterns in sexual experiences are at least in part socially constructed (Laan et al., 2021), it is critical to examine whether similar effects emerge in cultures with different gender norms and sexual scripts. Further, given the nature of our research question, some individuals were necessarily excluded from the analyses, including partnered individuals without available partner data, a group known to differ psychologically from dyadic samples (Park et al., 2021), as well as individuals who selectively declined to complete the sexual activity modules (see Supplementary material for comparisons between the analytic sample and participants excluded for this reason). Indeed, volunteer bias in sexuality research has been well documented (albeit largely among younger, college-aged samples; Dawson et al., 2019; Strassberg and Lowe, 1995; Wiederman, 1999), with individuals reporting more sexual experience and positive sexual attitudes being more likely to participate. If similar patterns extend to older populations, our findings, as well as much of the existing literature, may overestimate levels of sexual activity and disproportionately reflect experiences among those who are more (vs. less) sexually active. These considerations should be carefully accounted for when interpreting our results.

Further, although our prospective analyses adjusted for baseline levels of corresponding sexual experiences, allowing us to better capture changes, the discrepancy in reporting time frames across waves (referring to the past month vs. past year) may have influenced participants’ responses (Cameron & Santos-Iglesias, 2024), limiting the longitudinal interpretation of these analyses. Future research using equivalent measures across time points will allow for more accurate modeling of bidirectional relationships.

Finally, the scope of the sexual variables we assessed was constrained. The only specific activity we assessed was intercourse, but other specific forms of partnered sexual activities (e.g., caressing) could be more relevant to older adults and should be assessed. Further, equally important to successful sexual aging as the objective changes in one’s own and partner’s physical health conditions is the acceptance of and adaptation to those changes and subsequent ramifications in sexual experiences (Štulhofer, 2025). Assessing those experiences can provide deeper insights into the processes related to positive sexual aging.

In conclusion, these findings, while exploratory and small-to-modest in magnitude, underscore the importance of investigating dyadic effects and identifying objective health markers that may signal vulnerabilities to declines in sexual health. As sexual well being is increasingly recognized as a vital component of healthy aging, understanding the determinants of sexual experiences in later life warrants more systematic and inclusive research.

## Supplementary Material

gbag060_Supplementary_Data

## Data Availability

Authors have no right to share the data publicly. Information on how to access the data used in the current study can be found at https://www.elsa-project.ac.uk/accessing-elsa-data. This study was not preregistered.
