# Supplementary material for: Whose health matters? Longitudinal analyses of older romantic couples’ health, physical capabilities, and sexual experiences
Source: J Gerontol B Psychol Sci Soc Sci. 2026 Apr 3;81(5):gbag060. doi: 10.1093/geronb/gbag060 (PMC13129199; doi:10.1093/geronb/gbag060)
Supplement: gbag060_Supplementary_Data [file gbag060_supplementary_data.zip › JGSS suppl Park, Stenlund, & Steptoe.pdf]

***The Journals of Gerontology, Series B: Psychological Sciences and Social Sciences***
**Supplementary Material: Park, Stenlund, & Steptoe. Whose Health Matters? Longitudinal Analyses of Older Romantic Couples' Health, Physical Capabilities, and Sexual Experiences.**

**Table S1** below presents full statistics comparing individuals included in our analytic sample against partnered individuals who were excluded. Note that this includes people who were excluded because either they or their partner has not completed the sexual activity module (or any modules) at either wave. To address one reviewer's concern about selective non-response (i.e., not responding to the sexual activity module specifically), we also compared our analytic sample against a subset of individuals ($n$ = 1,211) who completed the self-completion questionnaire but *selectively* skipped the sexual activity module across waves. The results were very similar such that our sample was younger ($b$ = -3.24, $t$ = -9.23, $p$ < .001), had fewer health problems ($b$ = -0.15, $t$ = -3.27, $p$ < .001), and reported more frequent moderate and vigorous physical activity ($b$ = 0.09, $t$ = 2.46, $p$ = .01, and $b$ = 0.18, $t$ = 3.59, $p$ < .001). The two groups did not differ in their educational attainment, wealth, closeness with a partner, general life satisfaction, depressive symptoms, or frequency of light physical activity.

**Table S1.** Descriptive Statistics by Inclusion Status and Comparison Between Participants Included vs. Excluded from the Final Analyses

| ***Outcomes*** | **Included ($n$ = 2602)** | **Excluded ($n$ = 2492)** | ***Est.*** | ***SE*** | ***t*** | ***p*** |
|---|---|---|---|---|---|---|
| Age | 63.28 (8.09) | 64.87 (8.70) | -1.59 | 0.31 | -5.11 | < .001 |
| Closeness | 1.40 (1.23) | 1.54 (1.28) | 0.06 | 0.02 | 2.98 | .003 |
| Life satisfaction | 3.75 (0.51) | 3.70 (0.56) | 0.08 | 0.04 | 1.92 | .056 |
| Depression | 3.72 (0.74) | 3.66 (0.85) | -0.09 | 0.05 | -1.74 | .082 |
| Chronic health conditions | 5.31 (1.14) | 5.23 (1.20) | -0.14 | 0.04 | -3.60 | .001 |
| Light exercise | 3.47 (0.96) | 3.31 (1.08) | 0.06 | 0.02 | 2.47 | .014 |
| Moderate exercise | 0.99 (1.61) | 1.08 (1.67) | 0.16 | 0.03 | 5.00 | < .001 |
| Vigorous exercise | 2.16 (1.30) | 2.00 (1.27) | 0.16 | 0.04 | 3.85 | < .001 |
| | | | *OR* | *SE* | *z* | *p* |
| Tertiary education | 575 (24%) | 505 (22%) | 1.21 | 0.17 | 1.32 | .186 |
| Highest wealth tertile | 814 (33%) | 876 (34%) | 1.05 | 3.66 | 0.01 | .988 |

*Notes*. Means (with standard deviations in parentheses) are reported for continuous variables, and counts (with proportions in parentheses) are reported for categorical variables. Statistics are based on estimates (Est) and odds ratios (OR) from multilevel models accounting for clustering by couples.

**Table S2** corresponds to Table 3 in the main manuscript but examines the opposite direction of association (i.e., sexual experiences at baseline predicting health at follow-up). Because gait speed was not assessed at follow-up, these models are limited to self-rated health and grip strength. The significant actor sexual interest × gender interaction indicated that, for men, higher sexual interest predicted increases in grip strength at the follow-up ($b$ = 0.26, $t$ = 2.77, $p$ = .006), but this association was not significant for women ($b$ = -0.03, $t$ = -0.32, $p$ = .75).

**Table S2.** Self-Rated Health and Physical Capabilities Associated with Sexual Outcomes at Follow-up

| | **Interest** | | | **Activity** | | | **Intercourse** | | | **Satisfaction** | | |
|---|---|---|---|---|---|---|---|---|---|---|---|---|
| ***Predictors*** | ***b*** | ***SE*** | ***p*** | ***b*** | ***SE*** | ***p*** | ***b*** | ***SE*** | ***p*** | ***b*** | ***SE*** | ***p*** |
| | Outcomes: Self-rated health (Ns: 2588, 2590, 2566, 1474) | | | | | | | | | | | |
| Actor | 0.01 | 0.01 | .202 | 0.09 | 0.05 | .051 | -0.00 | 0.02 | .963 | -0.02 | 0.02 | .319 |
| Partner | 0.01 | 0.01 | .338 | 0.04 | 0.04 | .415 | 0.01 | 0.02 | .536 | -0.02 | 0.02 | .348 |
| | Outcomes: Grip strength (Ns: 1770, 1768, 1754, 976) | | | | | | | | | | | |
| Actor | 0.26 | 0.09 | .006 | 0.07 | 0.27 | .787 | 0.01 | 0.11 | .919 | -0.07 | 0.12 | .542 |
| Partner | 0.12 | 0.10 | .234 | 0.35 | 0.27 | .194 | 0.14 | 0.11 | .228 | 0.03 | 0.12 | .797 |
| Actor × Gender | -0.29 | 0.14 | .036 | | | | | | | | | |
| Partner × Gender | -0.18 | 0.14 | .188 | | | | | | | | | |

*Notes*. $b$ = unstandardized coefficient; $SE$ = standard error. All models adjusted for baseline levels of the outcome, as well as sociodemographic, relationship, and psychological covariates. Gender interactions were retained if the interaction with either the actor or partner effect was significant.

Tables S3-S10 present the full results from models examining the concurrent and prospective associations of self-rated health and physical capabilities with sexual experiences. In particular, Tables S6 and S10 report results from models treating gait speed as a continuous measure rather than categorizing it as slowness.

**Table Notes**

**Tables S3-S6**:

*Notes*. (A) = Actor effect; (P) = Partner effect. For categorical variables, the majority group served as a reference (i.e., White for race, upper secondary/vocational training for education, and married for marital status). For wealth, the second tertile was the reference group. Gender interactions were retained if the interaction with either the actor or partner effect was significant.

**Tables S7-S10**:

*Notes*. (A) = Actor effect; (P) = Partner effect. For categorical variables, the majority group served as a reference (i.e., White for race, upper secondary/vocational training for education, and married for marital status). For wealth, the second tertile was the reference group. Gender interactions were retained if the interaction with either the actor or partner effect was significant.

**Table S3.** Results From Models with Self-Rated Health Predicting Sexual Experiences at Baseline

| | Sexual interest | | | Activity | | | Intercourse | | | Satisfaction | | |
|---|---|---|---|---|---|---|---|---|---|---|---|---|
| *Predictors* | *Est.* | *SE* | *p* | *OR* | *SE* | *p* | *Est.* | *SE* | *p* | *Est.* | *SE* | *p* |
| Gender | -1.39 | 0.05 | <.001 | 0.11 | 0.03 | <.001 | -0.04 | 0.03 | .138 | 0.24 | 0.05 | <.001 |
| Age (A) | -0.05 | 0.00 | <.001 | 0.83 | 0.02 | <.001 | -0.03 | 0.00 | <.001 | -0.01 | 0.00 | .281 |
| Age (P) | -0.02 | 0.00 | <.001 | 0.92 | 0.02 | .002 | -0.03 | 0.00 | <.001 | 0.00 | 0.00 | .961 |
| Race (A) | -0.67 | 0.25 | .007 | 0.41 | 0.55 | .506 | -0.10 | 0.17 | .554 | 0.26 | 0.23 | .267 |
| Race (P) | -0.19 | 0.25 | .455 | 0.48 | 0.65 | .587 | 0.17 | 0.17 | .317 | 0.11 | 0.23 | .648 |
| Education (A) | | | | | | | | | | | | |
| < Upper sec. | -0.28 | 0.08 | <.001 | 0.45 | 0.18 | .052 | 0.02 | 0.06 | .762 | 0.17 | 0.08 | .038 |
| Tertiary | 0.14 | 0.07 | .048 | 1.35 | 0.71 | .573 | 0.08 | 0.06 | .161 | -0.06 | 0.07 | .357 |
| Education (P) | | | | | | | | | | | | |
| < Upper sec. | -0.09 | 0.08 | .269 | 0.57 | 0.23 | .168 | 0.08 | 0.06 | .190 | 0.10 | 0.08 | .209 |
| Tertiary | 0.26 | 0.07 | <.001 | 1.40 | 0.72 | .515 | 0.18 | 0.06 | .002 | -0.03 | 0.07 | .709 |
| Wealth ($1^{st}$) | -0.21 | 0.08 | .010 | 0.43 | 0.26 | .159 | -0.05 | 0.08 | .552 | 0.10 | 0.08 | .240 |
| Wealth ($3^{rd}$) | 0.09 | 0.08 | .251 | 1.64 | 1.10 | .460 | 0.24 | 0.08 | .004 | 0.00 | 0.08 | .995 |
| Marital status | 0.29 | 0.13 | .029 | 3.66 | 4.93 | .334 | 0.28 | 0.13 | .032 | 0.25 | 0.12 | .037 |
| Closeness (A) | 0.08 | 0.06 | .162 | 1.09 | 0.33 | .783 | 0.25 | 0.04 | <.001 | 0.49 | 0.07 | <.001 |
| Closeness (P) | 0.20 | 0.06 | .001 | 4.03 | 1.24 | <.001 | 0.24 | 0.04 | <.001 | 0.21 | 0.07 | .002 |
| Depression (A) | 0.03 | 0.02 | .129 | 1.11 | 0.13 | .353 | -0.02 | 0.02 | .284 | -0.07 | 0.02 | <.001 |
| Depression (P) | -0.00 | 0.02 | .906 | 0.89 | 0.10 | .328 | -0.02 | 0.02 | .289 | -0.01 | 0.02 | .676 |
| Health (A) | 0.17 | 0.04 | <.001 | 1.87 | 0.35 | .001 | 0.09 | 0.02 | <.001 | 0.17 | 0.04 | <.001 |
| Health (P) | -0.02 | 0.04 | .623 | 1.02 | 0.19 | .921 | 0.08 | 0.02 | .001 | -0.03 | 0.04 | .522 |
| Health (A) × Gender | -0.14 | 0.06 | .018 | | | | | | | -0.25 | 0.06 | <.001 |
| Health (P) × Gender | 0.08 | 0.06 | .173 | | | | | | | 0.14 | 0.06 | .021 |
| *N* | | 2595 | | | 2596 | | | 2584 | | | 1619 | |

**Table S4.** Results From Models with Grip Strength Predicting Sexual Experiences at Baseline

| | Sexual interest | | | Activity | | | Intercourse | | | Satisfaction | | |
|---|---|---|---|---|---|---|---|---|---|---|---|---|
| *Predictors* | *Est.* | *SE* | *p* | *OR* | *SE* | *p* | *Est.* | *SE* | *p* | *Est.* | *SE* | *p* |
| Gender | -1.24 | 0.12 | <.001 | 0.23 | 0.11 | .003 | -0.04 | 0.06 | .527 | 0.37 | 0.12 | .003 |
| Age (A) | -0.03 | 0.01 | <.001 | 0.84 | 0.03 | <.001 | -0.02 | 0.00 | <.001 | 0.00 | 0.01 | .766 |
| Age (P) | -0.03 | 0.01 | .001 | 0.90 | 0.03 | .002 | -0.03 | 0.00 | <.001 | -0.00 | 0.01 | .820 |
| Race (A) | -0.89 | 0.30 | .003 | 0.34 | 0.50 | .460 | -0.30 | 0.20 | .137 | 0.25 | 0.31 | .419 |
| Race (P) | -0.36 | 0.30 | .225 | 0.21 | 0.32 | .305 | 0.09 | 0.20 | .662 | 0.21 | 0.30 | .491 |
| Education (A) | | | | | | | | | | | | |
| < Upper sec. | -0.36 | 0.09 | <.001 | 0.41 | 0.19 | .049 | -0.00 | 0.07 | .988 | 0.08 | 0.10 | .419 |
| Tertiary | 0.17 | 0.09 | .052 | 1.51 | 0.91 | .500 | 0.09 | 0.07 | .192 | -0.04 | 0.09 | .672 |
| Education (P) | | | | | | | | | | | | |
| < Upper sec. | -0.14 | 0.09 | .120 | 0.37 | 0.17 | .030 | 0.07 | 0.07 | .308 | 0.05 | 0.10 | .596 |
| Tertiary | 0.24 | 0.09 | .005 | 1.43 | 0.85 | .550 | 0.15 | 0.07 | .032 | -0.07 | 0.08 | .440 |
| Wealth (1$^{st}$) | -0.26 | 0.10 | .007 | 0.38 | 0.25 | .142 | -0.14 | 0.10 | .164 | 0.09 | 0.10 | .352 |
| Wealth (3$^{rd}$) | 0.13 | 0.10 | .176 | 1.92 | 1.46 | .388 | 0.29 | 0.10 | .004 | -0.02 | 0.09 | .869 |
| Marital status | 0.35 | 0.19 | .067 | 2.64 | 4.43 | .563 | 0.48 | 0.19 | .012 | 0.36 | 0.18 | .043 |
| Closeness (A) | 0.08 | 0.07 | .234 | 0.98 | 0.35 | .965 | 0.24 | 0.05 | <.001 | 0.38 | 0.08 | <.001 |
| Closeness (P) | 0.19 | 0.07 | .006 | 3.93 | 1.40 | <.001 | 0.22 | 0.05 | <.001 | 0.09 | 0.08 | .273 |
| Depression (A) | -0.01 | 0.02 | .676 | 0.96 | 0.12 | .722 | -0.04 | 0.02 | .039 | -0.10 | 0.02 | <.001 |
| Depression (P) | 0.00 | 0.02 | .895 | 0.94 | 0.12 | .634 | -0.03 | 0.02 | .107 | -0.02 | 0.02 | .332 |
| Grip (A) | 0.03 | 0.01 | <.001 | 1.04 | 0.04 | .400 | 0.01 | 0.01 | .079 | 0.01 | 0.01 | .087 |
| Grip (P) | 0.00 | 0.01 | .988 | 0.98 | 0.04 | .650 | 0.01 | 0.01 | .093 | 0.00 | 0.01 | .462 |
| Grip (A) × Gender | -0.03 | 0.01 | .035 | | | | | | | | | |
| Grip (P) × Gender | 0.01 | 0.01 | .599 | | | | | | | | | |
| *N* | 1786 | | | 1783 | | | 1776 | | | 1060 | | |

**Table S5.** Results From Models with Slowness Predicting Sexual Experiences at Baseline

| | Sexual interest | | | Activity | | | Intercourse | | | Satisfaction | | |
|---|---|---|---|---|---|---|---|---|---|---|---|---|
| *Predictors* | *Est.* | *SE* | *p* | *OR* | *SE* | *p* | *Est.* | *SE* | *p* | *Est.* | *SE* | *p* |
| Gender | -1.41 | 0.08 | <.001 | 0.26 | 0.06 | <.001 | -0.06 | 0.04 | .120 | 0.20 | 0.08 | .018 |
| Age (A) | -0.04 | 0.01 | <.001 | 0.87 | 0.02 | <.001 | -0.02 | 0.01 | .009 | 0.02 | 0.01 | .163 |
| Age (P) | -0.01 | 0.01 | .189 | 0.96 | 0.03 | .108 | -0.02 | 0.01 | .001 | -0.01 | 0.01 | .611 |
| Race (A) | -0.65 | 0.40 | 0104 | 1.00 | 1.45 | .998 | -0.31 | 0.27 | .247 | 0.31 | 0.41 | .460 |
| Race (P) | -0.38 | 0.40 | .348 | 0.23 | 0.31 | .274 | 0.26 | 0.27 | .329 | 0.34 | 0.39 | .381 |
| Education (A) | | | | | | | | | | | | |
| < Upper sec. | -0.27 | 0.10 | .009 | 0.63 | 0.18 | .108 | 0.04 | 0.08 | .634 | 0.26 | 0.11 | .026 |
| Tertiary | 0.20 | 0.11 | .062 | 1.48 | 0.56 | .298 | 0.17 | 0.08 | .031 | -0.03 | 0.10 | .747 |
| Education (P) | | | | | | | | | | | | |
| < Upper sec. | -0.13 | 0.10 | .205 | 0.68 | 0.20 | .195 | 0.06 | 0.08 | .446 | 0.06 | 0.11 | .574 |
| Tertiary | 0.37 | 0.11 | .001 | 1.62 | 0.60 | .194 | 0.26 | 0.08 | .001 | 0.05 | 0.10 | .624 |
| Wealth ($1^{st}$) | -0.28 | 0.12 | .018 | 0.42 | 0.16 | .024 | -0.11 | 0.11 | .307 | -0.02 | 0.13 | .856 |
| Wealth ($3^{rd}$) | 0.14 | 0.11 | .211 | 1.64 | 0.66 | .217 | 0.31 | 0.10 | .003 | -0.09 | 0.11 | .419 |
| Marital status | 0.53 | 0.24 | .031 | 4.39 | 4.47 | .146 | 0.61 | 0.21 | .003 | 0.32 | 0.22 | .145 |
| Closeness (A) | -0.01 | 0.08 | .856 | 0.97 | 0.23 | .914 | 0.23 | 0.06 | <.001 | 0.48 | 0.10 | <.001 |
| Closeness (P) | 0.26 | 0.08 | .001 | 2.74 | 0.64 | <.001 | 0.21 | 0.06 | <.001 | 0.14 | 0.10 | .161 |
| Depression (A) | 0.04 | 0.03 | .138 | 1.10 | 0.09 | .274 | -0.01 | 0.02 | .661 | -0.07 | 0.03 | .016 |
| Depression (P) | -0.01 | 0.03 | .802 | 0.97 | 0.08 | .736 | -0.03 | 0.02 | .117 | -0.08 | 0.03 | .010 |
| Slow (A) | -0.54 | 0.19 | .004 | 0.55 | 0.19 | .089 | -0.02 | 0.09 | .818 | -0.04 | 0.14 | .784 |
| Slow (P) | 0.12 | 0.17 | .488 | 0.70 | 0.25 | .305 | -0.09 | 0.09 | .321 | 0.11 | 0.14 | .443 |
| Slow (A) × Gender | 0.50 | 0.25 | .048 | | | | | | | | | |
| Slow (P) × Gender | -0.52 | 0.25 | .039 | | | | | | | | | |
| *N* | 1401 | | | 1400 | | | 1392 | | | 744 | | |

**Table S6.** Results From Models with Gait Speed Predicting Sexual Experiences at Baseline

| | **Sexual interest** | | | **Activity** | | | **Intercourse** | | | **Satisfaction** | | |
|---|---|---|---|---|---|---|---|---|---|---|---|---|
| *Predictors* | *Est.* | *SE* | *p* | *OR* | *SE* | *p* | *Est.* | *SE* | *p* | *Est.* | *SE* | *p* |
| Gender | -1.41 | 0.08 | <.001 | 0.28 | 0.07 | <.001 | -0.05 | 0.04 | .162 | 0.19 | 0.08 | .022 |
| Age (A) | -0.04 | 0.01 | <.001 | 0.87 | 0.02 | <.001 | -0.01 | 0.01 | .015 | 0.02 | 0.01 | .181 |
| Age (P) | -0.01 | 0.01 | .183 | 0.95 | 0.03 | .050 | -0.02 | 0.01 | .001 | -0.00 | 0.01 | .686 |
| Race (A) | -0.68 | 0.40 | .090 | 0.84 | 1.18 | .900 | -0.33 | 0.27 | .225 | 0.33 | 0.41 | .429 |
| Race (P) | -0.43 | 0.40 | .287 | 0.23 | 0.30 | .256 | 0.27 | 0.27 | .312 | 0.32 | 0.39 | .405 |
| Education (A) | | | | | | | | | | | | |
| < Upper sec. | -0.28 | 0.10 | .007 | 0.62 | 0.18 | .106 | 0.04 | 0.08 | .608 | 0.26 | 0.12 | .026 |
| Tertiary | 0.20 | 0.11 | .063 | 1.48 | 0.57 | .307 | 0.17 | 0.08 | .034 | -0.03 | 0.11 | .751 |
| Education (P) | | | | | | | | | | | | |
| < Upper sec. | -0.14 | 0.10 | .179 | 0.67 | 0.20 | .185 | 0.06 | 0.08 | .457 | 0.07 | 0.11 | .555 |
| Tertiary | 0.37 | 0.11 | .001 | 1.59 | 0.59 | .213 | 0.26 | 0.08 | .001 | 0.05 | 0.11 | .646 |
| Wealth ($1^{st}$) | -0.29 | 0.12 | .016 | 0.39 | 0.15 | .015 | -0.12 | 0.11 | .285 | -0.01 | 0.13 | .926 |
| Wealth ($3^{rd}$) | 0.13 | 0.11 | .255 | 1.60 | 0.65 | .250 | 0.30 | 0.10 | .004 | -0.09 | 0.11 | .411 |
| Marital status | 0.49 | 0.24 | .045 | 4.09 | 4.16 | .166 | 0.60 | 0.21 | .004 | 0.32 | 0.22 | .139 |
| Closeness (A) | -0.02 | 0.08 | .848 | 0.97 | 0.23 | .913 | 0.23 | 0.06 | <.001 | 0.48 | 0.10 | <.001 |
| Closeness (P) | 0.27 | 0.08 | .001 | 2.72 | 0.64 | <.001 | 0.21 | 0.06 | <.001 | 0.14 | 0.10 | .160 |
| Depression (A) | 0.04 | 0.03 | .174 | 1.10 | 0.09 | .270 | -0.01 | 0.02 | .696 | -0.07 | 0.03 | .018 |
| Depression (P) | -0.01 | 0.03 | .716 | 0.95 | 0.08 | .578 | -0.04 | 0.02 | .097 | -0.07 | 0.03 | .016 |
| Speed (A) | 0.45 | 0.27 | .089 | 4.63 | 3.87 | .067 | 0.13 | 0.19 | .503 | 0.03 | 0.28 | .901 |
| Speed (P) | 0.18 | 0.27 | .486 | 0.68 | 0.57 | .643 | 0.06 | 0.20 | .758 | 0.01 | 0.28 | .958 |
| *N* | | 1401 | | | 1400 | | | 1392 | | | 744 | |

**Table S7.** Results From Models with Self-Rated Health Predicting Sexual Experiences at Follow-up

| | **Sexual interest** | | | **Activity** | | | **Intercourse** | | | **Satisfaction** | | |
|---|---|---|---|---|---|---|---|---|---|---|---|---|
| *Predictors* | *Est.* | *SE* | *p* | *OR* | *SE* | *p* | *Est.* | *SE* | *p* | *Est.* | *SE* | *p* |
| Gender | -0.61 | 0.05 | <.001 | 0.87 | 0.15 | .432 | -0.05 | 0.03 | .076 | 0.09 | 0.05 | .097 |
| Age (A) | -0.02 | 0.00 | <.001 | 0.96 | 0.02 | .021 | -0.01 | 0.00 | .019 | 0.00 | 0.00 | .480 |
| Age (P) | -0.01 | 0.00 | .102 | 0.97 | 0.02 | .075 | -0.01 | 0.00 | <.001 | -0.01 | 0.00 | .257 |
| Race (A) | -0.42 | 0.21 | .043 | 0.72 | 0.55 | 0.671 | 0.03 | 0.13 | .797 | -0.05 | 0.22 | .808 |
| Race (P) | 0.14 | 0.21 | .510 | 0.37 | 0.29 | .201 | -0.03 | 0.13 | .844 | 0.21 | 0.22 | .355 |
| Education (A) | | | | | | | | | | | | |
| < Upper sec. | -0.14 | 0.06 | .016 | 0.56 | 0.12 | .006 | -0.13 | 0.04 | .002 | 0.11 | 0.08 | .137 |
| Tertiary | 0.09 | 0.06 | .112 | 1.49 | 0.40 | .141 | 0.04 | 0.04 | .269 | -0.00 | 0.06 | .951 |
| Education (P) | | | | | | | | | | | | |
| < Upper sec. | -0.10 | 0.06 | .087 | 0.81 | 0.17 | .309 | -0.05 | 0.04 | .234 | 0.05 | 0.07 | .475 |
| Tertiary | 0.08 | 0.06 | .187 | 1.41 | 0.38 | .204 | -0.02 | 0.04 | .602 | -0.12 | 0.06 | .065 |
| Wealth (1st) | -0.04 | 0.06 | .444 | 0.93 | 0.22 | .749 | -0.02 | 0.05 | .710 | 0.12 | 0.07 | .089 |
| Wealth (3rd) | 0.09 | 0.06 | .101 | 1.37 | 0.35 | .227 | 0.06 | 0.05 | .199 | 0.07 | 0.06 | .246 |
| Marital status | -0.02 | 0.10 | .862 | 1.20 | 0.53 | .678 | 0.02 | 0.08 | .759 | -0.08 | 0.10 | .411 |
| Closeness (A) | 0.03 | 0.05 | .503 | 1.40 | 0.23 | .047 | 0.01 | 0.03 | .814 | 0.25 | 0.06 | <.001 |
| Closeness (P) | 0.03 | 0.05 | .576 | 1.27 | 0.22 | .157 | 0.08 | 0.03 | .013 | -0.05 | 0.06 | .387 |
| Depression (A) | 0.03 | 0.01 | .047 | 1.04 | 0.06 | .496 | 0.01 | 0.01 | .501 | -0.00 | 0.02 | .941 |
| Depression (P) | -0.00 | 0.01 | .881 | 1.03 | 0.06 | .570 | 0.01 | 0.01 | .467 | -0.03 | 0.02 | .081 |
| Baseline outcome | 0.57 | 0.02 | <.001 | 39.55 | 7.82 | <.001 | 0.58 | 0.01 | <.001 | 0.40 | 0.02 | <.001 |
| Health (A) | 0.12 | 0.03 | <.001 | 1.29 | 0.12 | .005 | 0.05 | 0.02 | .002 | 0.08 | 0.03 | .004 |
| Health (P) | 0.02 | 0.03 | .577 | 1.23 | 0.11 | .021 | 0.04 | 0.02 | .014 | 0.02 | 0.03 | .507 |
| Health (A) × Gender | -0.12 | 0.04 | .007 | | | | | | | | | |
| Health (P) × Gender | -0.03 | 0.04 | .526 | | | | | | | | | |
| *N* | 2566 | | | 2595 | | | 2570 | | | 1603 | | |

**Table S8.** Results From Models with Grip Strength Predicting Sexual Experiences at Follow-up

| | **Sexual interest** | | | **Activity** | | | **Intercourse** | | | **Satisfaction** | | |
|---|---|---|---|---|---|---|---|---|---|---|---|---|
| *Predictors* | *Est.* | *SE* | *p* | *OR* | *SE* | *p* | *Est.* | *SE* | *p* | *Est.* | *SE* | *p* |
| Gender | -0.46 | 0.10 | <.001 | 0.68 | 0.24 | .275 | -0.09 | 0.06 | .121 | 0.11 | 0.13 | .370 |
| Age (A) | -0.02 | 0.01 | .004 | 0.96 | 0.02 | .043 | -0.00 | 0.00 | .640 | 0.01 | 0.01 | .196 |
| Age (P) | -0.01 | 0.01 | .403 | 0.97 | 0.02 | .175 | -0.01 | 0.00 | .004 | -0.01 | 0.01 | .256 |
| Race (A) | -0.49 | 0.25 | .045 | 0.58 | 0.49 | .521 | -0.07 | 0.15 | .661 | -0.14 | 0.30 | .654 |
| Race (P) | 0.05 | 0.25 | .824 | 0.27 | 0.23 | .130 | 0.05 | 0.15 | .736 | 0.22 | 0.29 | .447 |
| Education (A) | | | | | | | | | | | | |
| < Upper sec. | -0.16 | 0.07 | .020 | 0.57 | 0.14 | .020 | -0.19 | 0.05 | <.001 | 0.08 | 0.09 | .392 |
| Tertiary | 0.16 | 0.07 | .020 | 1.66 | 0.52 | .104 | 0.03 | 0.05 | .479 | 0.01 | 0.08 | .931 |
| Education (P) | | | | | | | | | | | | |
| < Upper sec. | -0.06 | 0.07 | .414 | 0.77 | 0.19 | .280 | -0.04 | 0.05 | .382 | -0.02 | 0.09 | .840 |
| Tertiary | 0.07 | 0.07 | .305 | 1.35 | 0.41 | .318 | -0.00 | 0.05 | .964 | -0.10 | 0.08 | .209 |
| Wealth ($1^{st}$) | -0.05 | 0.07 | .437 | 0.67 | 0.18 | .130 | -0.01 | 0.06 | .871 | 0.05 | 0.08 | .531 |
| Wealth ($3^{rd}$) | 0.12 | 0.07 | .071 | 1.25 | 0.37 | .455 | 0.05 | 0.06 | .350 | 0.06 | 0.08 | .434 |
| Marital status | 0.22 | 0.14 | .099 | 1.50 | 0.91 | .502 | 0.05 | 0.11 | .643 | 0.19 | 0.15 | .195 |
| Closeness (A) | 0.08 | 0.06 | .173 | 1.31 | 0.25 | .160 | 0.01 | 0.04 | .815 | 0.25 | 0.08 | .002 |
| Closeness (P) | 0.02 | 0.06 | .720 | 1.17 | 0.23 | .418 | 0.04 | 0.04 | .338 | -0.09 | 0.08 | .239 |
| Depression (A) | 0.02 | 0.02 | .144 | 0.99 | 0.06 | .820 | 0.00 | 0.01 | .837 | -0.03 | 0.02 | .156 |
| Depression (P) | -0.02 | 0.02 | .157 | 0.98 | 0.06 | .723 | -0.01 | 0.01 | .361 | -0.03 | 0.02 | .221 |
| Baseline outcome | 0.59 | 0.02 | <.001 | 34.65 | 7.75 | <.001 | 0.58 | 0.02 | <.001 | 0.40 | 0.03 | <.001 |
| Grip (A) | 0.00 | 0.00 | .442 | 0.99 | 0.02 | .789 | 0.00 | 0.00 | .373 | -0.00 | 0.01 | .826 |
| Grip (P) | -0.00 | 0.00 | .770 | 1.01 | 0.02 | .448 | 0.02 | 0.01 | .001 | 0.00 | 0.01 | .612 |
| Grip (A) × Gender | | | | | | | 0.01 | 0.01 | .497 | | | |
| Grip (P) × Gender | | | | | | | -0.02 | 0.01 | .035 | | | |
| *N* | 1767 | | | 1782 | | | 1767 | | | 1049 | | |

**Table S9.** Results From Models with Slowness Predicting Sexual Experiences at Follow-up

| | Sexual interest | | | Activity | | | Intercourse | | | Satisfaction | | |
|---|---|---|---|---|---|---|---|---|---|---|---|---|
| *Predictors* | *Est.* | *SE* | *p* | *OR* | *SE* | *p* | *Est.* | *SE* | *p* | *Est.* | *SE* | *p* |
| Gender | -0.60 | 0.07 | <.001 | 1.04 | 0.21 | .849 | -0.08 | 0.04 | .032 | 0.24 | 0.09 | .007 |
| Age (A) | -0.02 | 0.01 | .004 | 0.98 | 0.02 | .370 | -0.01 | 0.00 | .142 | -0.00 | 0.01 | .761 |
| Age (P) | -0.01 | 0.01 | .305 | 0.97 | 0.02 | .140 | -0.01 | 0.00 | .014 | -0.01 | 0.01 | .185 |
| Race (A) | -0.57 | 0.32 | .080 | 0.40 | 0.35 | .302 | -0.01 | 0.20 | .971 | -0.23 | 0.38 | .540 |
| Race (P) | -0.08 | 0.31 | .796 | 0.25 | 0.23 | .128 | -0.06 | 0.20 | .764 | 0.21 | 0.35 | .550 |
| Education (A) | | | | | | | | | | | | |
| < Upper sec. | -0.16 | 0.07 | .034 | 0.56 | 0.12 | .009 | -0.13 | 0.05 | .013 | 0.10 | 0.10 | .328 |
| Tertiary | 0.20 | 0.08 | .012 | 1.47 | 0.44 | .190 | 0.07 | 0.05 | .220 | -0.00 | 0.09 | .991 |
| Education (P) | | | | | | | | | | | | |
| < Upper sec. | -0.06 | 0.07 | .393 | 0.88 | 0.20 | .564 | -0.05 | 0.05 | .377 | 0.03 | 0.10 | .748 |
| Tertiary | 0.07 | 0.08 | .398 | 1.25 | 0.36 | .436 | 0.05 | 0.05 | .332 | -0.09 | 0.09 | .335 |
| Wealth (1st) | 0.01 | 0.08 | .885 | 0.96 | 0.24 | .861 | 0.03 | 0.06 | .652 | 0.05 | 0.10 | .622 |
| Wealth (3rd) | 0.14 | 0.07 | .053 | 1.50 | 0.38 | .111 | 0.04 | 0.06 | .494 | -0.04 | 0.08 | .647 |
| Marital status | -0.09 | 0.16 | .582 | 1.79 | 1.10 | .345 | 0.09 | 0.13 | .499 | -0.03 | 0.16 | .859 |
| Closeness (A) | -0.04 | 0.06 | .518 | 1.34 | 0.25 | .112 | 0.00 | 0.04 | .923 | 0.27 | 0.09 | .004 |
| Closeness (P) | 0.07 | 0.06 | .240 | 1.06 | 0.20 | .755 | 0.02 | 0.04 | .598 | -0.08 | 0.09 | .382 |
| Depression (A) | 0.04 | 0.02 | .047 | 0.99 | 0.06 | .927 | 0.00 | 0.01 | .788 | -0.02 | 0.03 | .416 |
| Depression (P) | -0.01 | 0.02 | .752 | 1.03 | 0.06 | .609 | 0.00 | 0.01 | .847 | -0.02 | 0.03 | .395 |
| Baseline outcome | 0.59 | 0.02 | <.001 | 23.88 | 4.71 | <.001 | 0.58 | 0.02 | <.001 | 0.40 | 0.03 | <.001 |
| Slowness (A) | 0.03 | 0.09 | .741 | 0.70 | 0.18 | .178 | -0.00 | 0.06 | .960 | -0.31 | 0.18 | .086 |
| Slow (P) | 0.11 | 0.09 | .235 | 0.91 | 0.24 | .714 | -0.08 | 0.06 | .202 | 0.10 | 0.16 | .529 |
| Slow (A) × Gender | | | | | | | | | | 0.42 | 0.24 | .079 |
| Slow (P) × Gender | | | | | | | | | | -1.16 | 0.25 | <.001 |
| *N* | 1382 | | | 1399 | | | 1382 | | | 733 | | |

**Table S10.** Results From Models with Gait Speed Predicting Sexual Experiences at Follow-up

| | Sexual interest | | | Activity | | | Intercourse | | | Satisfaction | | |
|---|---|---|---|---|---|---|---|---|---|---|---|---|
| *Predictors* | *Est.* | *SE* | *p* | *OR* | *SE* | *p* | *Est.* | *SE* | *p* | *Est.* | *SE* | *p* |
| Gender | -0.58 | 0.07 | <.001 | 1.03 | 0.21 | .888 | -0.07 | 0.04 | .044 | 0.16 | 0.08 | .067 |
| Age (A) | -0.02 | 0.01 | .015 | 0.98 | 0.02 | .298 | -0.01 | 0.00 | .187 | -0.00 | 0.01 | .837 |
| Age (P) | -0.01 | 0.01 | .256 | 0.97 | 0.02 | .155 | -0.01 | 0.00 | .010 | -0.01 | 0.01 | .202 |
| Race (A) | -0.55 | 0.32 | .086 | 0.40 | 0.36 | .303 | -0.02 | 0.20 | .923 | -0.16 | 0.38 | .681 |
| Race (P) | -0.04 | 0.31 | .901 | 0.24 | 0.21 | .104 | -0.05 | 0.20 | .803 | 0.25 | 0.36 | .483 |
| Education (A) | | | | | | | | | | | | |
| < Upper sec. | -0.14 | 0.08 | .054 | 0.56 | 0.12 | .008 | -0.12 | 0.05 | .017 | 0.11 | 0.10 | .296 |
| Tertiary | 0.20 | 0.08 | .015 | 1.48 | 0.44 | .189 | 0.06 | 0.05 | .236 | 0.01 | 0.09 | .952 |
| Education (P) | | | | | | | | | | | | |
| < Upper sec. | -0.05 | 0.07 | .487 | 0.88 | 0.20 | .556 | -0.05 | 0.05 | .381 | 0.02 | 0.10 | .853 |
| Tertiary | 0.06 | 0.08 | .425 | 1.26 | 0.36 | .429 | 0.05 | 0.05 | .346 | -0.11 | 0.10 | .269 |
| Wealth (1st) | 0.04 | 0.08 | .597 | 0.92 | 0.22 | .735 | 0.03 | 0.06 | .683 | 0.05 | 0.10 | .652 |
| Wealth (3rd) | 0.13 | 0.07 | .067 | 1.49 | 0.38 | .119 | 0.04 | 0.06 | .536 | -0.03 | 0.08 | .767 |
| Marital status | -0.08 | 0.16 | .613 | 1.72 | 1.05 | .375 | 0.08 | 0.13 | .538 | -0.07 | 0.17 | .698 |
| Closeness (A) | -0.04 | 0.06 | .521 | 1.33 | 0.25 | .118 | 0.01 | 0.04 | .900 | 0.28 | 0.09 | .003 |
| Closeness (P) | 0.07 | 0.06 | .267 | 1.07 | 0.20 | .730 | 0.02 | 0.04 | .639 | -0.08 | 0.09 | .397 |
| Depression (A) | 0.05 | 0.02 | .014 | 0.99 | 0.06 | .815 | 0.00 | 0.01 | .743 | -0.02 | 0.03 | .487 |
| Depression (P) | -0.01 | 0.02 | .804 | 1.03 | 0.06 | .629 | 0.00 | 0.01 | .923 | -0.02 | 0.03 | .390 |
| Baseline outcome | 0.59 | 0.02 | <.001 | 24.02 | 4.74 | <.001 | 0.58 | 0.02 | <.001 | 0.39 | 0.03 | <.001 |
| Speed (A) | 0.47 | 0.20 | .019 | 1.16 | 0.73 | .808 | 0.08 | 0.13 | .545 | 0.28 | 0.35 | .418 |
| Speed (P) | -0.20 | 0.20 | .306 | 1.24 | 0.78 | .732 | 0.07 | 0.13 | .623 | 0.07 | 0.35 | .850 |
| Speed (A) × Gender | | | | | | | | | | -0.17 | 0.49 | .724 |
| Speed (P) × Gender | | | | | | | | | | 0.98 | 0.48 | .041 |
| *N* | 1382 | | | 1399 | | | 1382 | | | 733 | | |

**Figure S1**

Changes in Sexual Activity Across Waves

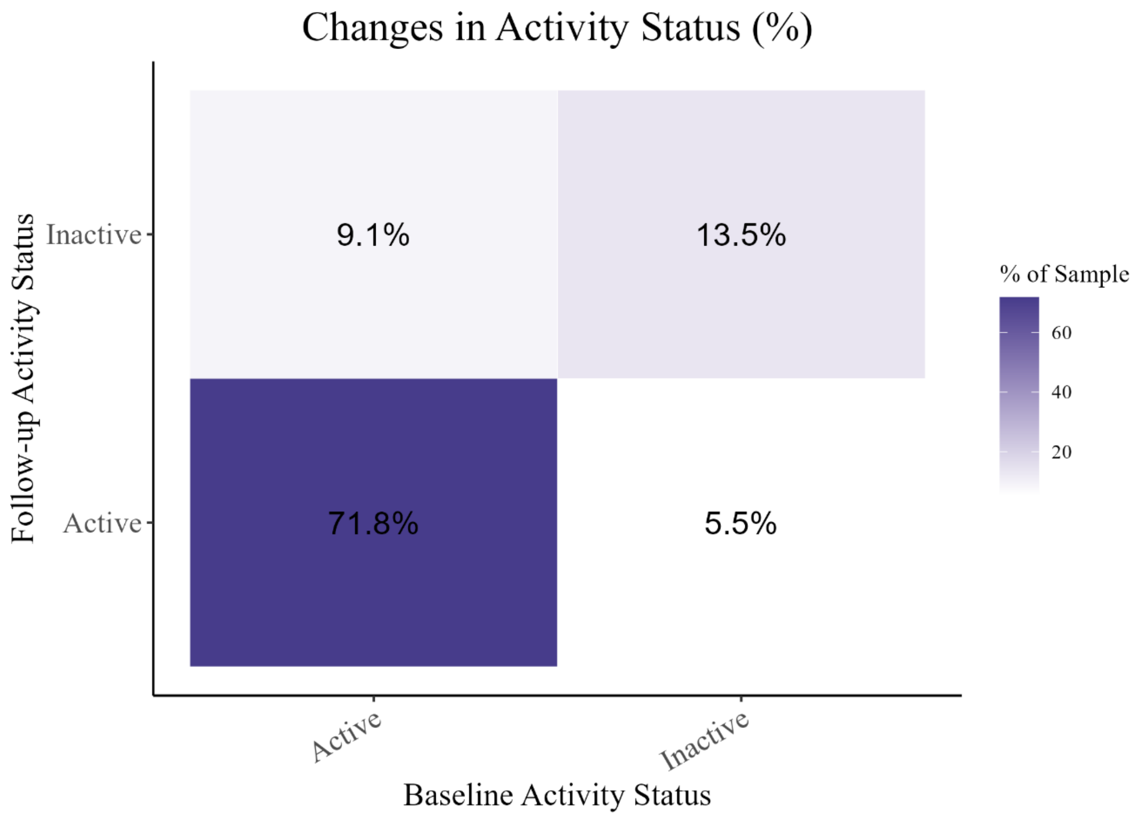


*Notes*. Each cell shows the percentage of the total sample falling into each transition category from baseline to follow-up.

**Figure S2**

Partner Agreement on Sexual Intercourse Frequency at Baseline

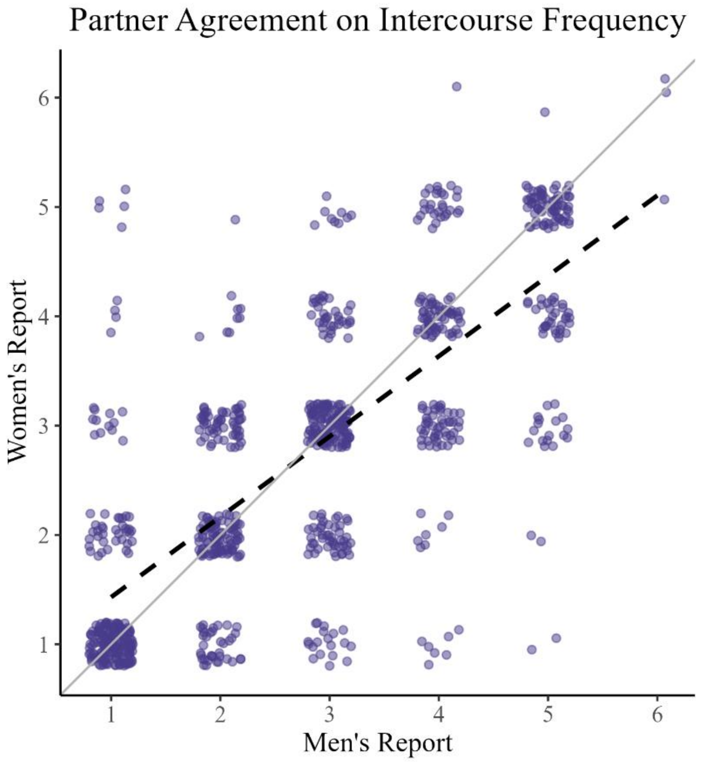


*Notes*. Responses to the question “How many times have you had or attempted sexual intercourse (vaginal, anal, or oral sex) during the past month?” are shown. Response options were: 1 (*not at all*), 2 (*once in the past month*), 3 (*2 or 3 times in the past month*), 4 (*once a week*), 5 (*2 or 3 times a week*), and 6 (*once a day or more*). The solid grey line represents perfect agreement, while the dashed line shows the best linear fit predicting women’s scores from men’s scores. Fifty-six percent of couples were in perfect agreement (i.e., on the solid line). The zero-order correlation between men’s and women’s reports was .74, indicating high rank-order similarity.
